# Multimorbidity in Large Canadian Urban Centres

**DOI:** 10.1093/eurpub/ckac129.294

**Published:** 2022-10-25

**Authors:** P Wilk, S Stranges, R Bellocco, T Bohn, H Samouda, K Nicholson, TT Makovski, A Maltby

**Affiliations:** 1 Department of Epidemiology and Biostatistics, Western University, London, Canada; 2 Institute of Social and Preventive Medicine, University of Bern, Bern, Switzerland; 3 Department of Precision Health, Luxembourg Institute of Health, Strassen, Luxembourg; 4 Department of Medical Epidemiology and Biostatistics, Karolinska Institutet, Solna, Sweden; 5 Department of Epidemiology, Maastricht University, Maastricht, Netherlands

## Abstract

**Introduction:**

Although health outcomes are related to the characteristics of the geographic areas in which people live, there is limited knowledge on how the prevalence of multimorbidity varies within and across major Canadian urban centres. Our goal was to assess the level of between-neighbourhood variation in the prevalence of multimorbidity in Canada's large urban centres, controlling for compositional effects associated with demographic and socioeconomic factors.

**Methods:**

Cross-sectional data from the 2015-2018 cycles of the Canadian Community Health Survey (CCHS) were used. Respondents (20 years and older) residing in one of the 35 census metropolitan areas (CMAs) were included (N = 100,803). Census tracts (CTs), relatively small and stable geographic areas nested within CMAs, were used as a measure of neighbourhood. To assess the between-neighbourhood differences in multimorbidity prevalence, we fitted sequential random intercept logistic regression models.

**Results:**

During the 2015-2018 period, 8.1% of residents of large urban centres in Canada had multimorbidity. The results from the unadjusted model indicate that 13.4% of the total variance in multimorbidity could be attributed to the between-neighbourhood differences. After adjustment for overall characteristics of the CMAs in which these neighbourhoods are located, as well as for individual-level demographic and socioeconomic factors related to compositional effects, 11.0% of the individual variance in multimorbidity could still be attributed to the between-neighbourhood differences.

**Discussion and Conclusions:**

There is significant and substantial geographic variation in multimorbidity prevalence across neighbourhoods in Canada's large urban centres. Residing in some neighbourhoods could be associated with increased odds of having multimorbidity, even after accounting for overall characteristics of the CMAs in which these neighbourhoods are located, as well as individual-level factors.

